# Resumption of Work by the War Crippled

**Published:** 1919-01

**Authors:** 


***On the Resumption of Work by the War Crippled.*** By J. Gourdon. *Bulletin de l'Académie de Médecine.* June 25, 1918.

The resuming of work by amputated and crippled men presents a scientific and economic interest. It would be, therefore, profitable to know the results obtained at the centers for re-education at Bordeaux.

The author's conclusions are based on 5014 cases, very serious, sometimes incurable. Out of that number 73% have been able to take up their professions again, with or without prothesis, and without special training. 27% had to be re-educated or professionally readapted.

It is interesting to note that only 30% of the wounded men were affected in the upper limbs.

The war-crippled man stands in quite a different light from the normal man; his physical and psychic powers are lessened, — the latter especially where will is concerned.

If the education and professional readaptation are to be really efficient, they must be preceded by a medico-surgical examination of the crippled man; his faculties, intelligence, energy and skill tested, and the work selected accordingly.

*Aptitudes of the Amputated and Crippled Men to go Back to Land Work*

Among the wounded men examined at the Bordeaux Center the percentage of farm hands and farmers was about 62%. It should be known that out of all classes of workmen, the land laborer is the one who takes up his work again the most easily, because

1. He loves his land.
2. He knows his work thoroughly.
3. Agricultural work does not necessitate any exact or precise movements.

It should be added that, owing to the great prothetic improvements made at the Bordeaux Center, 66 % of field laborers or proprietors took up their work again immediately.

The results observed are as follows :

Those with hand or forearm amputated give nearly the normal sum of work; those with the whole arm amputated, from 75% to 85%; those with disarticulation of the shoulder, from 40% to 50%.

Among men with lower limbs amputated, those amputated at the hip are decidedly the most handicapped, and accomplish but 50 o/o of the normal amount of work.

*Aptitudes of the War Crippled Men in Industrial Professions.*

Of course the work accomplished by mutilated men differs widely according to the class of work undertaken and the lesions of the men.

In basket-making, it varies from 50% to 75 % of the normal amount. In iron work from 40% to 50%.

In these observations, we consider,

1. Quality of execution
2. Quantity of work
3. Fatigue of the subject

All those who have taught the war crippled men are unanimous in asserting the very rapid progress of their pupils, whether commercial or administrative. This, because the men are more mature, keener and more attentive than primary school boys.

At the School for War Cripples at Bordeaux in nine months results were obtained that took sixteen months in the "Ecoles Professionnelles". All the pupils of that section were placed immediately and in excellent conditions.

*Conclusions* :

1. Most crippled men are fit for work again.
2. They should be educated in proper technical schools.
3. Education should start as soon as the wounds have healed, and a re-education prize ought to be added to the pension.
4. There is a decided advantage in reinstalling men in their previous occupation.

